# Adolescent undernutrition and early adulthood bone mass in an urbanizing rural community in India

**DOI:** 10.1007/s11657-015-0232-5

**Published:** 2015-09-01

**Authors:** Mika Matsuzaki, Hannah Kuper, Bharati Kulkarni, George B. Ploubidis, Jonathan C. Wells, Kankipati Vijaya Radhakrishna, Poornima Prabhakaran, Vipin Gupta, Gagandeep Kaur Walia, Aastha Aggarwal, Dorairaj Prabhakaran, K. V. Rameshwar Sarma, George Davey Smith, Yoav Ben-Shlomo, Sanjay Kinra

**Affiliations:** Department of Non-communicable Disease Epidemiology, London School of Hygiene and Tropical Medicine, Keppel Street, London, WC1E 7HT UK; Department of Clinical Research, London School of Hygiene and Tropical Medicine, Keppel Street, London, WC1E 7HT UK; National Institute of Nutrition, Indian Council of Medical Research Tarnaka, Jamai-Osmania, Hyderabad, 500 007 India; Department of Population Health and Statistics Centre for Longitudinal Studies, Institute of Education, University of London, 20 Bedford Way, WC1H 0AL London, UK; Childhood Nutrition Research Centre, UCL Institute of Child Health, 30 Guilford St, WC1N 1EH London, UK; Public Health Foundation of India, ISID Complex, 4 Institutional Area, Vasant Kunj, 110070 New Delhi, India; Department of Anthropology, University of Delhi, New Delhi, India; Centre for Chronic Disease Control, 4th Floor, Plot no. 47, Sector 44, Near Metro Huda Center, Gurgaon, Haryana, 122002 India; MRC Integrative Epidemiology Unit, School of Social and Community Medicine, University of Bristol, BS8 2BN Bristol, UK; School of Social and Community Medicine, Canynge Hall, 39 Whatley Road, BS8 2PS Bristol, UK

**Keywords:** Undernutrition, Adolescence, Bone mineral density, Longitudinal

## Abstract

**Summary:**

The long-term effects on bone health of nutritional status in adolescence are unclear. The impact of adolescent and current body mass on bone mass in young adulthood in rural India was assessed. Current lean mass was a more important determinant of bone mass than thinness during adolescence in this population.

**Purpose/introduction:**

Adolescence is a crucial period for skeletal growth. However, the long-term effects on bone health of nutritional status in adolescence, particularly in the context of nutritional transition, are unclear. The current manuscript assessed the impact of adolescent and current body size on bone mass in young adulthood in an Indian rural community that is undergoing rapid socioeconomic changes.

**Methods:**

The Andhra Pradesh Children and Parents Study is a prospective cohort study in Hyderabad, India. In 2003–2005, the study collected anthropometric and cardiovascular data on adolescents (mean age = 16 years old). The second and third waves of the study in 2009–2012 collected data on current anthropometric measures, areal bone mineral density (aBMD) in hip and lumbar spine (L1–L4) measured by dual-energy X-ray absorptiometry, and living standards of the trial participants who were now young adults (mean age = 22 years old).

**Results:**

The median body mass index (BMI) of the 722 participants included in this analysis was 16.8 kg/m^2^ during adolescence, while the median BMI as young adults was 19.3 kg/m^2^. Lower aBMD during adulthood was associated with lower adolescent BMI (β (95 % confidence interval) for hip aBMD 0.017 (0.013 to 0.022) and LS aBMD 0.012 (0.008 to 0.016)). This association was attenuated upon adjustment for current fat and lean mass (β (95 % CI) for hip aBMD 0.00 (−0.005 to 0.005) and LS aBMD 0.005 (0.000 to 0.01)). There was clear evidence for positive associations between aBMDs and current lean mass.

**Conclusions:**

Current lean mass was a more important determinant of bone mass than thinness during adolescence in this population. Weight gain during late adolescence and young adulthood coupled with improvement in lean mass may help to mitigate any adverse effects that pre-adulthood undernutrition may have on bone mass accrual.

## Introduction

Suboptimal peak bone mass is associated with higher risk of osteoporotic fractures in later life [1, 2]. Studies from high income countries have shown that 90 % of peak bone mass is accrued before age 18 in healthy individuals [1, 3, 4]. Skeletal growth during adolescence is therefore an important determinant of peak bone mass. Large body size, high level of weight-bearing physical activity, and adequate micronutrient intake are some of the key determinants of bone mass accrual [1].

Undernutrition is commonly observed in low and middle income countries (LMICs). In India, the prevalence of undernutrition remains high although it has been slowly declining over the last 2 decades [5, 6]. As a result, some young adults who experienced undernutrition during childhood and adolescence have attained at least normal body mass index as adults [7].

A number of studies have suggested positive associations between adult bone mass and birthweight as well as weight during infancy [8–10]. On the other hand, association between peak bone mass and thinness during adolescence has not been adequately studied in lean populations from LMICs. Some studies from high income countries examined longitudinal effects of anorexia nervosa during adolescence and showed that successful recovery from anorexia nervosa may mitigate some of the negative effects of low body weight during adolescence [11, 12]. Since adolescence is a crucial period for skeletal growth, it is important to understand whether undernutrition during adolescence has any long-term effects on bone mass.

While studies have generally found a positive association between body mass and bone mass, fat and lean mass may be differently associated with bone mass [13–15]. Lean mass is influenced by both diet and physical activity level. Weight-bearing physical activity during adolescence is associated with higher bone mass [4, 16]. It is therefore important to understand how gains in overall weight, fat mass, and lean mass may contribute to skeletal development in young adults who experienced nutritional transition during adolescence.

The Andhra Pradesh Children and Parents Study (APCAPS) is a prospective cohort study from southern India. The study community has been experiencing nutritional transition due to urbanization over the past decade. The current manuscript assessed whether being underweight during adolescence is associated with lower peak bone mass in young adults, some of whom have experienced improvements in nutritional status since adolescence.

## Methods

### Study design

The analyses in this study used data from three waves of data collection (2003–2005, 2009–2010, and 2010–2012) of the APCAPS study, established through long-term follow up of the Hyderabad Nutrition Trial (HNT). The HNT studied impact of the Integrated Child Development Services (ICDS) scheme, a national community outreach program providing food supplementation along with health, hygiene, and nutrition education, immunization, anemia control, and basic health care to pregnant and lactating women and children under the age of 6 years [17].

### Initial trial (1987–90) and the first wave of data collection (W1: 2003–5)

A detailed description of the initial trial (HNT) and the first wave of data collection (the first follow-up of the HNT) have previously been published [18]. Briefly, a controlled “stepped wedge design” study was conducted in 1987–1990, using the opportunity afforded by the incremental expansion of ICDS program. A total of 29 villages in two adjacent administrative areas near Hyderabad city in India were selected, one with ICDS program already in place (15 intervention villages) and the other awaiting implementation (14 control villages). In the intervention villages, a nutritional supplement made of corn-soya blend and soybean oil was available daily to all pregnant and lactating women and children under 6 years. The meal (upma) contained, on average, 2.09 MJ and 20–25 g protein for pregnant and lactating women and about 1.25 MJ and 8–10 g protein for children under 6 years old. The supplementation was associated with a small but statistically robust (61 g; 95 % CI 18 to 104 g; *p*=0.007) increase in the birth weight of the offspring [18]. During the first wave of data collection in 2003–2005, 1165 adolescents aged 13–18 years who were still resident in these villages were reexamined [18]. The adolescents in the intervention villages were 14 mm (95 % CI 4 to 23 mm; *p* = 0.007) taller and had more favorable measures of insulin resistance and arterial stiffness as shown by a 20 % (95 % CI 3 to 39 %; *p* = 0.02) lower homoeostasis model assessment score, which describes levels of insulin resistance, and 3.3 % (95 % CI 1 to 5.7 %; *p* = 0.008) lower augmentation index.

### The second and third waves of data collection (W2/3: 2009–2012)

Since the second and third waves of data collection were conducted within a relatively short period of time (2009–2012), the analyses in this manuscript combined data from these two waves of data collection (W2/3). W2/3 examined markers for chronic diseases affecting cardiovascular, musculoskeletal, and mental health. All consenting participants underwent DXA measurements at the National Institute of Nutrition (NIN), Hyderabad, and physical measurements at NIN (W2) or the village clinics (W3). In cases where participants attended both waves of data collection, the data from the third wave were used, unless there were artifacts in DXA scans from the W3, which prompted the use of data from W2.

The present analyses were restricted to participants from the first wave of data collection who also underwent DXA scans during W2/3.

### Measurements

#### Questionnaire data (W1/2/3)

A semi-structured questionnaire was administered to all participants by a trained interviewer. A subset of questions (14/29) from the Standard of Living Index (SLI) in the National Health Family Survey-2, a summary measure of household level asset-based scale devised for Indian surveys, was used to estimate socioeconomic position, as joint family structures are common in rural India [19]. We collected information on the quality of house, toilet facilities, source of lighting and drinking water, ownership of clock, radio, television, bicycle, motorcycle, car, refrigerator, telephone, and agricultural land. These items were weighted to give a maximum score of 34, using weights developed by the International Institute of Population Science in India [19]. Education was classified in four levels: no formal education, primary (1 to 4 standard), secondary (5 to 12 standard), and beyond secondary level education. Current tobacco use was defined as smoking, chewing, or snuffing tobacco in the last 6 months.

#### Puberty (W1)

Four puberty stages were set based on sexual maturation on the basis of time since the onset of menstruation (girls) and testicular volume (boys) [20]. The boys assessed testicular volume in private, using Prader’s orchidometer with volumes ranging from 1 to 25 ml. This self-assessment was validated against measurements by clinicians in an external sub-study [20].

#### Anthropometric data (W1/2/3)

Weight was measured to the nearest 0.1 kg with a digital SECA balance, and standing height was measured to the nearest 1 mm with a plastic stadiometer (Leicester height measure). Measurements were taken twice, and the average of two values was used in the analysis (coefficients of variation for height 0.67 %; weight 0.09 %). Body mass index (BMI) was calculated as weight (kg)/height (m^2^). Cutoff points of BMI ≤17.0 and ≤18.5 were used for underweight in adolescence and adulthood respectively [21, 22].

#### DXA scanning (W2/3)

Bone mass measurements were assessed with DXA on a Hologic Discovery A model. The whole body scan was performed with the participant supine on the scanning bed with their arms resting by their sides. On the basis of repeated measurements for 30 participants with Hologic Discovery A, the coefficients of variation were determined to be 0.7 % for hip bone mineral density (BMD), 1.3 % for LS aBMD, and 0.9 % for whole-body aBMD. Women suspected of pregnancy were excluded from DXA scanning, and the scans were taken only after confirming the negative pregnancy by conducting urine pregnancy test. Standard Hologic software options were used to define regions of the body (head, arms, trunk, and legs). Scans were coded for artifacts by a visual inspection, and those with major movement as well as incomplete scans were excluded from the present analyses. For lumbar spine (LS) scans, pathological changes such as osteoarthritis affecting two or more vertebrae were excluded; if only one vertebra was affected, the scan was reanalyzed after the affected part was excluded [23]. Areal bone mineral density (aBMD in g/cm^2^) was calculated from bone mineral content (BMC in g) and bone area (BA in cm^2^) for total hip and lumbar spine (L1–L4). Fat and lean mass indexes (FMI and LMI) were based on fat and lean mass (kg) from whole-body scans/height (m^2^). Major movements were counted as artifacts and removed from analysis with fat and lean mass.

### Statistical analysis

Descriptive statistics were calculated for each sex. The associations of hip and lumbar spine BA, BMC, and aBMD with adolescent body size were modeled in multilevel regression models that accounted for village clusters (29 villages). FMI was log-transformed because it had a positively skewed distribution. Complete-case analysis was used.

Four models were fitted for each of the two outcome variables (hip and LS aBMD): Model 1 assessed each of the explanatory variables (adolescent BMI, current (young adulthood) BMI, current FMI, current LMI), adjusting for sex, age at W1, age at W2/3, height at W1 (cm), and height at W2/3 (cm). Model 2 examined the association between aBMD and adolescent BMI, adjusting for current BMI, age at W1, age at W2/3, sex, height at W1, and height at W2/3. In model 3, current BMI in model 2 was replaced with current FMI and LMI. Model 4 replaced current BMI in model 2 with conditional BMI to examine the effect of change in BMI, by using residuals from a regression model in which current BMI was regressed on adolescent BMI. We also adjusted model 3 for other potential confounders such as puberty stages at W1, adolescent height, current height, adolescent SLI, current SLI, and current tobacco use. Interaction terms between sex and adolescent BMI, current FMI, current LMI, and change in BMI were examined in models 3 and 4. There was weak evidence for an interaction with current FMI for LS aBMD, where the effect of FMI was slightly higher in female (the interaction term *β*: 0.08; *p* = 0.04). Residuals from the multilevel models were checked for normality (Shapiro-Wilk test) and heteroscedasticity and found reasonably normally distributed and homoscedastic at each level.

All analyses were conducted using *R*, version 3.0.0, and multilevel modeling was done with nlme version 3.1-109.

### Quality control

We produced detailed protocols and regularly checked compliance to standardize the work of the fieldwork team. The anthropometric equipment was calibrated at the start of every clinic. The aBMD estimation process was automated in software, which reduces the potential for bias arising from the DXA technician. Hip and LS DXA scans were analyzed by a single trained technician. For quality assurance of DXA scans, a spine phantom was scanned every day to check for acceptable ranges.

### Ethics statement

The study received approvals from the ethics committees of the NIN (Hyderabad, India), the Indian Council of Medical Research (ICMR), Centre for Chronic Disease Control, and London School of Hygiene and Tropical Medicine (London, UK). Approval was also sought from the village heads and their panchayats in each of the 29 villages. Written informed consent or witnessed thumbprint if illiterate was obtained from the participants prior to their inclusion in the study.

## Result

Of the 1165 participants of the first wave of data collection (W1), 722 participants (62 %) had their height and weight measured both as adolescents and as young adults and also underwent DXA scans during the second and third waves of data collection (W2/3). Of those, scans without major artifacts were available in 710 (98 %) participants for hip aBMD and 715 (99 %) for lumbar spine aBMD. Eighty-seven percent of the participants also had scans without major artifacts for whole-body estimation of fat and lean mass. Information on the other variables including SLI and occupation was available for ≥99 % of the participants.

Table 1 summarizes the key characteristics of the participants. Fifty-six percent of the participants were underweight (BMI < 17) during adolescence. The participants were still lean as young adults although the percentage of underweight individuals (BMI ≤ 18.5) decreased to 38 %. A majority of the participants were students during W1, but only a third of the participants were students during W2/3. Women were more likely to be unemployed and engaged in household work as young adults. Both hip and lumbar spine aBMD values were generally lower than the reference values for the Indian population (Table 2) [24]. Underweight adults (BMI ≤ 18.5) showed lower aBMD than others with normal BMI (data not shown).

**Table 1:** Characteristics of the subjects who participated in the Andhra Pradesh Parents and Children Study both in 2003–2005 (W1) and in 2009–2012 (W2/3)

	Women			Men		
	*n* ^c^	W1	W2/3	*n* ^c^	W1	W2/3
Age (year)	220	15.8 (1)	22 (1.3)	502	15.9 (0.9)	21.8 (1.3)
Height (cm)	220	151.2 (5.9)	153.1 (5.6)	502	158.5 (8.7)	167 (6.2)
Weight (kg)	220	40.6 (6)	45.9 (8.2)	502	41.9 (7.5)	56.0 (9.4)
Body mass index (kg/m^2^)	220	17.7 (2.2)	19.6 (3.2)	502	16.6 (1.9)	20.1 (3)
Fat mass index (kg/m^2^)	216	*n/a*	5.8 (2)	499	*n/a*	3.4 (1.6)
Lean mass index (kg/m^2^)	216	*n/a*	13.1 (1.5)	499	*n/a*	15.9 (1.8)
SLI	217	12.9 (5)	18.1 (4.7)	499	10.6 (4.3)	18.7 (4.3)
Occupation (%)	220			502		
Student^b^		84.2	27.3		85.5	34.3
Employed		15.8	32.3		12	61.2
Neither		11.7	40.5		2.6	4.6
Tobacco use (*n*)^a^	215			500		
Current		0	2		1	85
Former		0	0		0	3
Never		215	220		499	415

All values are mean (sd) unless otherwise noted

*W1* the first wave of data collection (2003–2005), *W2/3* the second and third waves of data collection (2009–2012), *SLI* standard of living index, *n/a* not available

^a^Current tobacco use included smoking, chewing, or snuffing tobacco in the last 6 months; former users stopped using tobacco products 6 months ago or more

^b^Student for W1 included one man who worked and studied at the same

^c^All *n* were same for W1 and W2/3 except that SLI was available for 219 women in W2/3. Tobacco use information was available for 222 women in W2/3

**Table 2:** Mean hip and lumbar spine bone mass of participants of the Andhra Pradesh parents and children study in 2009–2012 (W2/3)

Total hip	Women		Men	
	*n*	Mean (sd)	*n*	Mean (sd)
BA (cm^2^)	217	28.43 (2.59)	493	35.83 (3.44)
BMC (g)	217	23.92 (3.98)	493	34.25 (5.36)
BMD (g/cm^2^)	217	0.837 (0.096)	493	0.952 (0.115)
Lumbar spine				
BA (cm^2^)	216	48.81 (4.89)	499	57.56 (5.63)
BMC (g)	216	42.76 (8.02)	499	54.68 (8.99)
BMD (g/cm^2^)	216	0.869 (0.104)	499	0.945 (0.105)

*BA* bone area, *BMC* bone mineral content, *BMD* bone mineral density

Positive association between current aBMD and adolescent BMI was attenuated upon adjustment for current BMI in hip, but the association remained for lumbar spine (Tables 3 and 4). There was no strong evidence of interactions between the change in BMI between W1 and W2/W3 and adolescent BMI (data not shown). In model 3, current lean mass index was strongly associated with current aBMD, whereas there was no strong evidence for association between current aBMD and adolescent BMI or current fat mass index. Model 4 showed positive associations between current aBMDs and conditional BMI. Adjustment for other potential confounders (puberty stages at W1, adolescent height, current height, adolescent SLI, current SLI, and current tobacco use) did not materially change the results.

**Table 3:** Multivariable models examining associations between body mass index during adolescence (2003–2005) and current bone mineral density (2009–2012) in hip in young adults of the Andhra Pradesh children and parents study (2003–2012)

	Hip BMD							
	Model 1		Model 2		Model 3		Model 4	
	*β*		*β*		*β*		*β*	
	(95 % CI)	*p*	(95 % CI)	*p*	(95 % CI)	*p*	(95 % CI)	*p*
Adolescent BMI	0.017	*<*0.001	0.003	0.32	0.00	0.97	0.015	*<*0.001
	(0.013 to 0.022)		(−0.003 to 0.008)		(−0.005 to 0.005)		(0.011 to 0.019)	
Current BMI	0.015	*<*0.001	0.014	*<*0.001				
	(0.012 to 0.017)		(0.01 to 0.017)					
Current FMI	0.087	*<*0.001			−0.018	0.23		
	(0.062 to 0.113)				(−0.047 to 0.011)			
Current LMI	0.03	*<*0.001			0.032	*<*0.001		
	(0.026 to 0.034)				(0.026 to 0.038)			
Conditional BMI^a^	0.015	<0.001					0.014	*<*0.001
	(0.012 to 0.019)						(0.01 to 0.017)	

Conditional BMI was estimated from current BMI regressed on adolescent BMI

Model 1 is a base model examining association between BMD and each of four explanatory variables (adolescent BMI, adulthood BMI, adulthood fat mass, and adulthood lean mass), adjusting for sex, age at the first wave of data collection (W1) in 2003–2005 (adolescence), age at the second and third waves (W2/3) in 2009–2012 (current/adulthood), height at W1 (cm), and height at W2/3 (cm)

Model 2 examined association between adolescent BMI (kg/cm^2^) and adulthood BMD (g/cm^2^) adjusting for current BMI, sex, age at W1, age at W2/3, height at W1, and height at W2/W3

Model 3 examined association between adolescent BMI and adulthood BMD, adjusting for current FMI (kg/m^2^), current LMI (kg/m^2^), sex, age at W1, and age at W2/W3, height at W1, and height at W2/W3

Model 4 examined association between adolescent BMI and adulthood BMD, adjusting for conditional BMI, sex, age at W1, age at W2/3, height at W1, and height at W2/W3

*BMI* body mass index (kg/m^2^), *FMI* fat mass index (kg/m^2^, log-transformed), *LMI* lean mass index (kg/m^2^)

**Table 4:** Multivariable models examining associations between body mass index during adolescence (2003–2005) and current bone mineral density (2009–2012) in the lumbar spine in young adults of the Andhra Pradesh children and parents study (2003–2012)

	LS BMD							
	Model 1		Model 2		Model 3		Model 4	
	*β*		*β*		*β*		*β*	
	(95 % CI)	*p*	(95 % CI)	*p*	(95 % CI)	*p*	(95 % CI)	*p*
Adolescent BMI	0.012	*<*0.001	0.006	0.03	0.005	0.06	0.011	*<*0.001
	(0.008 to 0.016)		(0.001 to 0.011)		(0.00 to 0.01)		(0.007 to 0.015)	
Current BMI	0.008	*<*0.001	0.006	*<*0.001				
	(0.006 to 0.011)		(0.003 to 0.009)					
Current FMI	0.048	*<*0.001			−0.013	0.41		
	(0.024 to 0.072)				(−0.043 to 0.017)			
Current LMI	0.016	*<*0.001			0.014	*<*0.001		
	(0.012 to 0.02)				(0.008 to 0.02)			
Conditional BMI^a^	0.007	*<*0.001					0.006	*<*0.001
	(0.004 to 0.01)						(0.003 to 0.009)	

Conditional BMI was estimated from current BMI regressed on adolescent BMI

Model 1 is a base model examining association between BMD and each of four explanatory variables (adolescent BMI, adulthood BMI, adulthood fat mass, and adulthood lean mass), adjusting for sex, age at the first wave of data collection (W1) in 2003–2005 (adolescence), age at the second and third waves (W2/3) in 2009–2012 (current/adulthood), height at W1 (cm), and height at W2/W3 (cm)

Model 2 examined association between adolescent BMI (kg/cm^2^) and adulthood BMD (g/cm^2^) adjusting for current BMI, sex, age at W1, age at W2/3, height at W1, and height at W2/W3

Model 3 examined association between adolescent BNU and adulthood BMD, adjusting for current FMI (kg/m^2^), current LMI (kg/m^2^), sex, age at W1, and age at W2/3, height at W1, and height at W2/W3

Model 4 examined association between adolescent BMI and adulthood BMD, adjusting for conditional BMI, sex, age at W1, age at W2/3, height at W1, and height at W2/W3

*LS* lumbar spine, *BMI* body mass index (kg/m^2^), *FMI* fat mass index (kg/m^2^, log-transformed), *LMI* lean mass index (kg/m^2^)

## Discussion

There were less underweight young adults from this transitional rural community compared to during adolescence. Although these young adults on average had low bone mass, there was no clear evidence for an association between bone mass during young adulthood and thinness during adolescence when adjusted for current fat and lean mass. There was stronger evidence for a positive association between bone mass and lean mass than fat mass in young adulthood.

### Comparison with previous research

While there are a number of studies examining association between bone mass in later life and birthweight, there are relatively few studies focusing on the long-term effects of nutritional status during adolescence on adult bone mass [8]. The New Delhi Birth Cohort examined the associations between bone mass during adulthood (age 33–39) and early life height and weight [25]. This cohort also had low BMI (15.1 kg/m^2^ for boys and 15.4 kg/m^2^ for girls) at age 11, although by age 33–39, the average BMI had increased to above 25. The study found positive associations between femoral neck and lumbar spine BMC and aBMD during adulthood and BMI at age 11, but similarly to our findings, these associations were attenuated upon adjustment for adult BMI. They also assessed changes in BMI in infancy, childhood, and adolescence and found that the change in BMI during adolescence was most strongly associated with adulthood bone mass. In our previous analyses, we found no strong evidence for a positive association between areal bone mineral density as young adults and early life nutritional supplementation [26].

The study subjects in the APCAPS gained weight between late adolescence and young adulthood. The Penn State Young Women’s Health Study compared healthy women who gained weight in late adolescence (17–22 years) to those who had stable weight [27]. Those who gained weight had higher aBMD and greater bone cross sectional area in proximal femur shaft. This result is in line with our findings where larger gain in BMI during young adulthood is associated with higher aBMDs. Of note, their study also found that the bone strength index decreased in women who became overweight during late adolescence and suggested a potential negative effect of excess weight gain during adolescence on bone strength.

Studies have suggested different patterns of associations between aBMD and fat and lean mass [14, 28–30]. In our study, there was no strong evidence for positive associations between aBMDs and fat mass. On the other hand, there was more consistent evidence for a positive association between aBMD and lean mass than fat mass, similarly to previous studies assessing relative contributions of fat and lean mass to bone mass accrual [28, 29].

It is important to note that aBMD in this study population was generally lower than values for bone mass values for young adults reported in a national DXA study in India [25]. The individuals with current BMI in the normal range had aBMD values closer to the national reference values than those with lower BMI values [25]. The current study found stronger evidence for an association between bone mass in young adulthood and current BMI than adolescent BMI. The weight gain during late adolescence may not have been sufficient for some of the study participants to achieve full catch-up growth. It is also possible that weight-bearing physical activity level was not high enough during late adolescence and young adulthood in this population.

### Strengths and limitations

The main strength of this study is the availability of longitudinal data on height and weight, allowing the assessment of long-term effects of undernutrition during adolescence on bone mass in young adulthood. The study subjects experienced a unique circumstance where nutritional status of the study subjects improved greatly toward the end of the skeletal growth phase due to socioeconomic development in their villages. This setting allowed for an assessment of potential mitigation of the effects of undernutrition in early adolescence through improved nutritional status in late adolescence and young adulthood. Another strength of the study was the use of fat and lean mass from DXA scans to understand how different types of body mass may be distinctly associated with bone mass.

The study also had some limitations. The DXA measurements were not performed during adolescence, making it less clear whether and how bone mass improved, as weight, fat mass, and lean mass increased during adolescence. However, the association between body size and bone mass has been shown repeatedly in previous studies [14, 27], and therefore, it is reasonable to assume that the study subjects who were mostly underweight during adolescence also had lower z-scores for bone mass for their age than healthier adolescents. Finally, due to a lack of detailed nutritional and activity data from W1, we could not explore long-term effects of lifestyle risk factors during adolescence that may have been important for skeletal growth.

## Conclusions

In healthy individuals, much of bone mass accrual occurs during adolescence. As socioeconomic development continues in low- and middle-income countries, many children and adolescents are experiencing the effects of nutritional transition. Our findings suggest that weight gain combined with improvement in lean mass in young adulthood may be able to help mitigate adverse effects of undernutrition during adolescence on bone mass in young adulthood.
